# The use of ultrasound in primary care: longitudinal billing and cross-sectional survey study in Switzerland

**DOI:** 10.1186/s12875-020-01209-7

**Published:** 2020-07-01

**Authors:** Dima Touhami, Christoph Merlo, Joachim Hohmann, Stefan Essig

**Affiliations:** 1 Department of Health Sciences and Medicine, University of Lucerne, 6002 Lucerne, Switzerland; 2 Instiute of Primary and Community Care, 6004 Lucerne, Switzerland; 3 Swiss Paraplegic Research, 6207 Nottwil, Switzerland; 4 Medical Faculty, University of Basel, 4056 Basel, Switzerland; 5 Institute of Radiology and Nuclear Medicine, Cantonal Hospital Winterthur, 8400 Winterthur, Switzerland; 6 Swiss Society of Ultrasound in Medicine, 5000 Aarau, Switzerland

**Keywords:** Ultrasound, General practice, Primary care, General practitioners, Clinical indications, Ultrasound productivity, Switzerland

## Abstract

**Background:**

Ultrasound imaging is utilized in Swiss primary care; however, little is known regarding the extent to which it is performed. With this study, we aim to (1) provide an overview of ultrasound use by general practitioners (GPs), and (2) determine the clinical indications of ultrasound in Swiss general practice.

**Methods:**

This is a quantitative study, analyzing 15 years of billing data from 213 GPs in Central Switzerland, and cross-sectional survey data completed by 61 GPs attending 26 certification and refresher courses offered by the Swiss Society of Ultrasound in Medicine (SGUM).

**Results:**

According to billing data, 49% of the GPs used ultrasound and provided 130,245 exams to 67,180 patients between 2004 and 2018. Over the years, ultrasound use became more frequent among GPs. Male GPs provide more ultrasound exams than female GPs. Patients that are female, ≥65 years, and multi-morbid had more ultrasound exams compared to males, patients < 65 years, and those with only one morbidity, respectively. GPs provided a mean of 129 ultrasound exams per physician-year. Abdominal ultrasound comprised almost 69% of all exams. According to survey data, indications covered many organ systems and clinical conditions, with abdominal indications being most frequent among them.

**Conclusions:**

The use of ultrasound is high among general practitioners and it covers a wide range of clinical indications. Ultrasound is utilized primarily in the diagnosis of clinical indications of the abdomen, and more often for female than male patients.

## Background

In the last two decades, the use of ultrasound by non-radiologists has rapidly expanded to emerge as a standard imaging modality of modern clinical practice in many countries [1–4]. The increasing demands for imaging in the community and outside hospital settings, the relative simplicity of the ultrasound technique, the low cost of the equipment, and the reported improvement of ultrasound’s durability and portability are all equally reflected in its growing popularity in primary care settings [2–8]. Even more, a growing body of literature acclaims ultrasound as a useful tool in primary care for the detection of acute conditions, management of chronic diseases, and for procedural guidance [2, 4, 7, 9]. The safety, noninvasiveness, faster diagnosis, reduction in medical referrals, and the psychological reassurance of patients are additional aspects that contribute to the diagnostic value of ultrasound in this setting [2, 7, 10].

The use of ultrasound in general practice seem to be different between countries. The percentage of primary care physicians using ultrasound varies between 1 and 67% across European countries. Further, access to in-house ultrasonography is shown to be significantly associated with larger practices, and the availability of a qualified colleague with ultrasonography experience [11]. In Switzerland, the utilization of ultrasound for abdominal exams in general practice is known to be established for many years; however, research on its use is sparse. One study conducted across primary care in the French-speaking part of Switzerland, indicated that the demand for scanning was low and abdominal ultrasound comprised most of ultrasound requests [1]. More so, none of the primary care physicians in the study received training in ultrasound or had equipment in their offices. Thus, the knowledge deficit regarding the efficacy of ultrasound and the clinical indications of its use were implied as reasons behind this low demand [1]. Another study assessed the use and the organizational aspects of point of care ultrasound (POCUS) in general practice in 12 European countries including Switzerland. Results of the study illustrated that the clinical indications for applying POCUS in primary care were similar among all countries, especially for musculoskeletal/joints, abdominal, urogenital, cardiac and vessels. However, obstetrical and gynecological exams were not conducted by GPs in Switzerland, Austria and Catalonia. In addition, certification in ultrasound was found necessary for the remuneration of exams in Switzerland, Germany, Netherlands and Austria. Nevertheless, lack of time and training were main barriers for utilizing ultrasound in the 12 countries [12].

To our knowledge, no studies have thoroughly examined the clinical indications of ultrasound in Swiss general practice, the purposes of its use and the frequency of its utilization. For this reason, establishing a basic understanding of the ultrasound uses and the extent of uses in Swiss primary care is deemed crucial. Therefore, the aim of this study is to provide an overview on the utilization of ultrasound by GPs, and to determine the clinical indications of ultrasound in Swiss general practice.

## Methods

### Study design

Data from two groups of GPs were included in the study to answer our research questions. The billing group comprised of ultrasound-certified GPs practicing in Central Switzerland. The survey group included GPs attending ultrasound SGUM certification and recertification courses.

### Data collection and study population

#### Longitudinal billing data

Billing data of GPs was provided through a trust center (medkey AG, Luzern, Switzerland) that collects, analyzes and evaluates the performance of physicians in Central Switzerland for benchmark comparisons. Since data comprised sensitive billing information, GPs’ consent was pre-requisite. A total of 401 physicians in Central Switzerland on contract with the trust center were invited to share their data that had been collected between January 2004 and September 2018. The extracted GP data included their basic demographic information including age, sex, area of expertise, practice location, practice size, the total number of consultations, stability in their data reporting, and the contract start date with the trust center. We determined the urban level of their practice location in accordance with the European Degree of Urbanization 2011 [13]. We applied the cantonal registry of physicians to complement any missing characteristics of the GPs.

The extracted patient data included all patients with any ultrasound exam during the above-mentioned period. Since ultrasound exams for pediatric population comprised only 3% of overall total, age was restricted to patients 18 years and older. Patient data included their age, sex, number of doctor visits, type of ultrasound examination, and morbidity. Types of ultrasound exams were based on the Swiss ambulatory reimbursement codes of services (Tarmed), accounting for changes in the codes between 2004 and 2018 [14]. We applied the Pharmacy-based Cost Group (PCG) model to determine the morbidity of patients based on the Anatomical Therapeutic Chemical Classification System (ATC Code) of their medication [15, 16]; patients with two or more morbidities were considered multi-morbid [17].

#### Cross-sectional survey data

We conducted a cross-sectional study with a questionnaire survey, completed by GPs attending ultrasound courses offered by SGUM. The questionnaires were provided in German and French. With the approval of SGUM, we disseminated paper-based questionnaires in bulk to course coordinators supervising 26 certification and refresher courses for abdominal ultrasound and the first POCUS courses occurring between September 2018 and January 2019 anywhere in Switzerland. Based on the SGUM website, 796 participants were estimated to attend the 26 courses. However, we had no beforehand information on the medical specialty and number of GPs attending. For this reason, we provided written instructions to course coordinators requesting them to distribute questionnaires to participating GPs. We included GPs with past or current experience with ultrasound, or those planning any future use of ultrasound in their practices. We collected their basic demographic and professional information, including age, sex, practice type and address, professional titles, and certifications in ultrasound. GPs were asked to identify the clinical indications for which they presently utilize or anticipate using ultrasound for adult patients. An extensive list of indications was created based on previous surveys and published literature [7, 8, 18–20]. Additionally, we asked the GPs about the purposes and reasons for use, relevance of ultrasound to their practices, and comfort with their skills to utilize ultrasound for the clinical assessment of patients and procedural guidance.

### Data analysis

The billing data provided an overview of ultrasound use in Swiss primary care, including the prevalence of ultrasound use by GP and patient characteristics, ultrasound productivity, and types of ultrasound. Ultrasound productivity refers to the frequency of ultrasound usage (that is, daily, weekly, or less often), and the annual number of ultrasound exams per physician-year. The frequency was determined according to the average workload of primary care physicians in Switzerland [21]. Physician-years were calculated based on the contract duration of GPs with the trust center, and the consistency in data reporting. Yearly ultrasound productivity includes GPs that performed at least one ultrasound examination in the respective year. Overall ultrasound productivity includes all GPs who performed at least one ultrasound across all years.

Complementarily to billing data, the survey data are about detailed clinical indications of ultrasound, and views of GPs on various aspects of ultrasound use.

We report relative frequencies and mean scores with corresponding 95% confidence intervals. Survey instruments were designed utilizing the SoSci survey software (SoSci Survey GmbH, Munich, Germany) for data collection. The billing and survey data were both imported to Stata/SE 15.1 (StataCorp LLC, College Station, USA) for data management and analysis.

## Results

### Response

For billing data, 213 out of 401 GPs (53% response rate), consented to participate in our study. The survey data represented GPs (*n* = 61) who were attending the SGUM courses, of which 89% were practicing in German-speaking regions of Switzerland. The surveyed GPs participated in a variety of twelve ultrasound certification courses and two refresher courses. Of these courses, eight presented abdominal ultrasound and six covered POCUS. Due to the data collection methodology, we could not determine the response rate of GPs in the survey group. Table 1 summarizes the characteristics of the GPs who participated in our study.

**Table 1:** Characteristics of general practitioners stratified by sex, age group and practice location, type of practice and frequency of US use

	Billing Group			Survey Group			Overall
	Users	Non-users	Total	Current^a^	Non-users	Total	
**Overall**	**104**	**109**	**213**	**54**	**7**	**61**	**274**
**Sex**	**104**	**109**	**213**	**50**	**7**	**57**	**270**
Male, n (%)	84 (81)	75 (69)	159 (159)	29 (58)	2 (29)	31 (54)	190 (70)
Female, n (%)	20 (19)	34 (31)	54 (25)	21 (42)	5 (71)	26 (46)	80 (30)
**Age group**	**104**	**109**	**213**	**52**	**6**	**58**	**271**
< 40, n (%)	12 (12)	10 (9)	22 (10)	35 (67)	2 (33)	37 (64)	59 (20)
≥ 40, n (%)	92 (88)	99 (91)	191 (90)	17 (33)	4 (67)	21 (36)	212 (80)
**Practice location**	**104**	**109**	**213**	**53**	**6**	**59**	**272**
Urban, n (%)	25 (24)	35 (33)	60 (28)	21 (40)	1 (17)	22 (37)	82 (30)
Suburban, n (%)	60 (58)	51 (46)	111 (52)	26 (49)	5 (83)	31 (53)	142 (52)
Rural, n (%)	19 (18)	23 (21)	42 (20)	6 (11)	0	6 (10)	48 (18)
**Practice type**	**104**	**109**	**213**	**54**	**6**	**60**	**273**
Individual, n (%)	35 (34)	44 (40)	79 (37)	12 (22)	1 (17)	13 (22)	92 (33)
Group, n (%)	69 (66)	65 (60)	134 (63)	32 (59)	3 (50)	35 (58)	169 (62)
General Outpatient, n (%)	–	–	–	4 (7)	1 (17)	5 (8)	5 (2)
*Permanence,* n (%)	–	–	–	6 (11)	1 (17)	7 (12)	7 (3)

A dash (−) denotes that there were no observations under this group

^a^Currently using ultrasound

### Prevalence of ultrasound use

Of 213 GPs in the billing group, 49% (*n* = 104) were utilizing ultrasound in their practices. Compared to non-users, GPs who used ultrasound were more often male and working in suburban areas, slightly younger and more often working in group practices (Table 1).

Table 2 comprises the characteristics of 67,180 patients obtained from the billing data. Over the 15 years, patients had a mean of 1.94 ultrasound exams during 19.6 doctor visits. Male patients (43%, *n* = 28,552) had more doctor visits (20.66), but less ultrasound exams (1.88) than female patients did. Patients 65 years and older (29%, *n* = 19,499) had most doctor visits (28.28) and most exams (2.06). Multi-morbid patients (63%, *n* = 42,010) had more doctor visits (27.23) and more ultrasound exams (2.10) than patients with single morbidity (Table 2). Among patients with single morbidity (17%, *n* = 11,399), patients with thyroid disorders (2.37%, *n* = 270) had most ultrasound exams (2.27) among all morbidities (see Supplementary Table 1).

**Table 2:** The characteristics of patients, doctor visits and ultrasound exams per patient, over 15 years

	Patients	Doctor visits		US exams
	n (%)	Mean per patient (CI)	n (%)	Mean per patient (CI)
**Overall**	**67′180**	**19.63**	**130′245**	**1.94 (1.93–1.95)**
**Sex**				
Male	28′552 (43)	20.66 (20.41–20.92)	53′796 (41)	1.88 (1.86–1.90)
Female	38′628 (57)	18.86 (18.63–19.09)	76′449 (59)	1.98 (1.96–1.99)
**Age (years)**				
18–39	18′564 (28)	12.58 (12.36–12.79)	34′600 (27)	1.86 (1.84–1.89)
40–64	29′117 (43)	18.33 (18.09–18.57)	55′437 (42)	1.90 (1.89–1.92)
≥ 65	19′499 (29)	28.28 (27.89–28.67)	40′208 (31)	2.06 (2.04–2.09)
**Morbidity**				
1 morbidity	11′399 (17)	8.80 (8.64–8.96)	19′356 (15)	1.70 (1.67–1.72)
> 1 morbidity	42′010 (63)	27.23 (26.99–27.46)	88′345 (68)	2.10 (2.08–2.12)
Unknown morbidity	13′771 (20)	4.97 (4.86–5.08)	22′544 (17)	1.64 (1.62–1.66)

*US* Ultrasound, *CI* Confidence Interval at 95% significance level

### Ultrasound productivity

Table 3 summarizes the ultrasound productivity of GPs in the billing group. The ultrasound productivity of GPs (*n* = 8) with missing contract details with the trust center were excluded from this analysis. In the fifteen-year period, GPs (*n* = 96) had a range of between 0.07 and 1256 ultrasound exams per physician year, and provided an overall mean of almost 129 ultrasound exams per physician year. Although statistically insignificant, the mean was higher among female GPs (131), and those GPs practicing in urban areas (167; see Table 3).

**Table 3:** The association of GP characteristics with ultrasound productivity^a^

	GPs n (%)	Mean of ultrasound exams per physician year (CI)^a^
**Overall**	**96**	128.72 (94.17–163.27)
**Sex**		
Male	80 (83)	128.20 (99.09–157.30)
Female	16 (17)	131.32 (32.09–294.74)
**Practice location**		
Urban	21 (22)	166.84 (35.53–298.15)
Suburban	56 (58)	114.86 (85.97–143.74)
Rural	19 (20)	127.44 (54.09–200.79)

*CI* Confidence interval at 95% significance level

^a^ Ultrasound productivity: number of exams per physician year

**Fig. 1:**
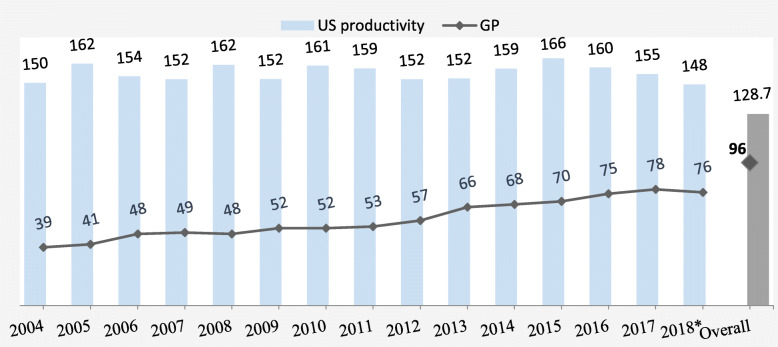
Annual ultrasound productivity and number of GPs offering ultrasound between 2004 and 2018. US: ultrasound; US productivity refers to the annual number of ultrasound exams per physician-year. * 2018 data is up to 30 September 2018. The yearly values are based on those GPs that performed at least one ultrasound examination in the respective year. The overall values are based on all GPs, i.e., they performed at least one ultrasound across all years

Figure 1 shows an increase in the number of GPs performing ultrasound from 39 to 78 between 2004 and 2017. The mean of yearly ultrasound productivity of GPs had a range of between 148 and 166 exams (see Supplementary Table 2). Ten GPs (10%) were performing ultrasound on a daily basis, 39 GPs (41%) at least weekly and 47 GPs (49%) less often.

### Types of ultrasound

Of all exams (*n* = 130,245), most were abdominal scans (68%, *n* = 89′124), performed by almost all ultrasound users (97%, *n* = 101; see Table 4). Among female patients, second most common type of examination was denoted as “rectal and/or vaginal endosonography” (27%, *n* = 20,394) performed by 25% of users (*n* = 26). Among male patients, the second most common type was “musculoskeletal and soft parts conditions” (10%, *n* = 5242). Across the 15 years, the majority of patients (87%, *n* = 58,312) had one type of ultrasound, 11% (*n* = 7795) had two types, and the remaining 2% of patients (*n* = 1073) had up to five different types of ultrasound exams.

**Table 4:** Distribution of ultrasound exams by type, stratified by patient sex, and frequency of GPs performing it, over 15 years

	Frequency of ultrasound exams Patients			GPs ^**a**^
Type of ultrasound	*Females* *n* = 76′449 n (%)	*Males* *n* = 53′796 n (%)	Total *n* = 130′245 n (%)	*n* = 104 n (%)
Head and neck	4′399 (5.75)	1′791 (3.33)	6′190 (4.75)	66 (63.46)
Breast and axilla	248 (0.32)	7 (0.01)	255 (0.20)	13 (12.50)
Abdomen^b^	42′984 (56.23)	46′140 (85.77)	89′124 (68.43)	101 (97.12)
Vessels with Doppler	500 (0.65)	500 (0.93)	1000 (0.77)	30 (28.85)
Musculoskeletal and soft parts	5′222 (6.83)	5′242 (9.74)	10′464 (8.03)	66 (63.46)
Rectal and/or vaginal endosonography	20′394 (26.68)	46 (0.09)	20′440 (15.69)	26 (25.00)
Pregnancy related	2′484 (3.25)	–	2′484 (1.91)	18 (17.31)
Interventional	218 (0.29)	70 (0.13)	288 (0.22)	14 (13.46)

A dash (−) denotes that there were no observations under this group

^**a**^Total percentage does not add to 100, many GPs performed more than one type. Ultrasound type is organized according to Tarmed classification

^b^Abdominal ultrasound includes urogenital and transabdominal exams

### Clinical indications

Table 5 shows the clinical uses of ultrasound as reported by the survey group. A total of 54 GPs reported frequent use of ultrasound for abdominal clinical conditions, predominantly free fluid in the abdomen (93%, *n* = 50), kidney congestion (93%, *n* = 50), bladder filling condition (91%, *n* = 49), cholecystolithiasis (91%, *n* = 49), liver disease management (89%, *n* = 48), splenomegaly (89%, *n* = 48), and cholecystitis (85%, *n* = 46). Other indications target cardiac (*n* = 34), vascular (*n* = 32), thoracic (*n* = 45), musculoskeletal and other conditions (*n* = 43), prostate and testicular evaluation (*n* = 42; see Table 5). Ultrasound for women’s reproductive health was indicated by 26% of the GPs (*n* = 14) in the survey group; ovarian pathology (*n* = 12), uterine assessment (*n* = 11), and abnormal vaginal bleeding (*n* = 6) were among the main obstetric and gynecological conditions indicated for ultrasound use (See Table 5).

**Table 5:** Clinical indications of ultrasound for current and future use

	Present use	Anticipated use	
		Current users	Non-users
Total	*n* = 54	*n* = 25	*n* = 7
**Abdomen**^a^	**54 (100)**	**16 (64)**	**7 (100)**
*Liver disease assessment,* n (%)	48 (89)	2 (13)	4 (57)
*Splenomegaly,* n (%)	48 (89)	2 (13)	6 (86)
*Pancreas assessment,* n (%)	38 (70)	4 (25)	4 (57)
*Cholecystolithiasis,* n (%)	49 (91)	4 (25)	7 (100)
*Cholecystitis,* n (%)	46 (85)	6 (38)	6 (86)
*Obstructive jaundice,* n (%)	42 (78)	6 (38)	4 (57)
*Free fluid in the abdomen,* n (%)	50 (93)	4 (25)	7 (100)
*Renal colic,* n (%)	44 (81)	5 (31)	5 (71)
*Kidney congestion,* n (%)	50 (93)	4 (25)	5 (71)
*Appendicitis*, n (%)	36 (67)	6 (38)	3 (43)
*Diverticulitis*, n (%)	30 (56)	5 (31)	4 (57)
*Hernia*, n (%)	24 (44)	6 (38)	2 (29)
*Abdominal aortic aneurysm*, n (%)	42 (78)	6 (38)	5 (71)
*Abdominal trauma*, n (%)	33 (61)	2 (13)	5 (71)
*Irritable bowel disease*, n (%)	18 (33)	4 (25)	2 (29)
*Bladder diseases*, n (%)	37 (69)	4 (25)	5 (71)
*Bladder filling condition*, n (%)	49 (91)	2 (13)	7 (100)
*Evaluation of the prostate*, n (%)	40 (74)	3 (19)	4 (57)
*Testicular torsion*, n (%)	16 (30)	4 (25)	1 (14)
*Testicular pain*, n (%)	24 (44)	3 (19)	1 (14)
*Testicular inflammation*, n (%)	20 (37)	3 (19)	1 (14)
**Cardiac**^a^	**34 (63)**	**11 (44)**	**6 (86)**
*Pericardial effusion*, n (%)	33 (97)	4 (36)	6 (100)
*Evaluation of ventricular size and function*, n (%)	4 (12)	8 (73)	1 (17)
*Undifferentiated hypotension*, n (%)	6 (18)	6 (55)	–
*Valvular assessment*, n (%)	2 (6)	5 (45)	1 (17)
**Vascular**^a^	**32 (59)**	**13 (52)**	**1 (14)**
*Deep venous thrombosis*, n (%)	28 (88)	8 (62)	1 (100)
*Intima-media thickness / Carotid plaque*, n (%)	17 (53)	4 (31)	–
*Jugular venous distention,* n (%)	5 (16)	4 (31)	1 (100)
**Thoracic**^a^	**45 (83)**	**15 (60)**	**6 (86)**
*Pneumothorax,* n (%)	19 (42)	10 (67)	3 (50)
*Pleural effusion,* n (%)	42 (93)	5 (33)	4 (67)
*Ribs fractures,* n (%)	25 (56)	10 (67)	5 (83)
*Chest pain****OR****Shortness of breath****OR****Dyspnea*, n (%)	11 (24)	5 (33)	1 (17)
*Auscultatory Changes in the lungs,* n (%)	9 (20)	7 (47)	1 (17)
*Chest trauma,* n (%)	14 (31)	6 (40)	3 (50)
**Women’s Reproductive Health**^a^	**14 (26)**	**3 (12)**	**–**
*Ovarian pathology,* n (%)	12 (86)	3 (100)	–
*First term pregnancy,* n (%)	5 (36)	2 (67)	–
*Ruptured ectopic pregnancy,* n (%)	3 (21)	2 (67)	–
*Abnormal vaginal Bleeding,* n (%)	6 (43)	2 (67)	–
*Uterine ultrasound****OR****uterine mass assessment,* n (%)	11 (79)	2 (67)	–
**Musculoskeletal & Other conditions**^a^	**43 (80)**	**17 (68)**	**3 (43)**
*Fracture,* n (%)	12 (28)	8 (47)	2 (67)
*Joint effusion, tendon or ligament rupture,* n (%)	21 (49)	6 (35)	3 (100)
*Joint assessment and muscular injuries,* n (%)	16 (37)	5 (29)	–
*Evaluation of rheumatoid arthritis,* n (%)	4 (9)	3 (18)	1 (33)
*Thyroid****OR****Parathyroid,* n (%)	31 (72)	9 (53)	1 (33)
*Adrenal screening,* n (%)	8 (19)	4 (24)	–
*Evaluation of lymph nodes****OR****Palpable neck mass,* n (%)	33 (77)	5 (29)	–
*Abscesses and cysts,* n (%)	38 (88)	4 (24)	2 (67)
*US for guided procedures*, n (%)	14 (33)	–	–
*US for pain management*, n (%)	4 (9)	–	–

^a^ Overall number of GPs using and/or anticipating using ultrasound for respective bodily systems

A total of 32 GPs in the survey group anticipated utilizing ultrasound for other conditions. Of these, 25 (78%) GPs were current users and anticipated using ultrasound for additional clinical indications, including pneumothorax (*n* = 10), rib fractures (*n* = 10), deep venous thrombosis (*n* = 8), and abdominal aortic aneurysm (*n* = 6; see Table 5).

### General views

Supplementary Table 3 presents the views of surveyed GPs on the use of ultrasound. Of all users (*n* = 53), about 91% (*n* = 48) identified faster diagnosis and earlier medical intervention, standard of practice (77%, *n* = 41), the availability of a machine (68%, *n* = 36) and patients’ comfort and convenience (57%, *n* = 30) as general reasons behind using ultrasound. All current users reported utilizing ultrasound for diagnostic purposes, about 94% (*n* = 51) reported ultrasound skills as somewhat (*n* = 24) to very relevant (*n* = 27) to their practices. About 81% (*n* = 44) stated to be somewhat (*n* = 24) to very comfortable (*n* = 20) to use ultrasound for clinical assessment. Only 30% (*n* = 16) indicated using ultrasound for guided procedures.

A total of seven GPs in the survey group reported no current usage of ultrasound but specified their intent for future application. Faster diagnosis and earlier medical intervention (83%, *n* = 5) were identified as main reasons for the planned use. All GPs (*n* = 7) anticipated utilizing ultrasound for diagnostic purposes (see Supplementary Table 3).

## Discussion

### Main findings

The findings of this study suggest that about half of the GPs utilize ultrasound in practices of Central Switzerland, female patients receive more ultrasound exams compared to males and that ultrasound is primarily applied in the diagnosis of indications of the abdomen, but also includes many other indications. Despite an increasing frequency of GPs performing ultrasound throughout the 15 years, the mean of ultrasound productivity of GPs remained stable.

### Interpretation & comparison to existing literature

Although statistically insignificant, the results from our study show variation in the use of ultrasound productivity. Our findings show that less female GPs use ultrasound, yet seem to have a higher ultrasound productivity on average. This finding is important; it may signal a rise in the use of ultrasound due to the growing number of female physicians in primary care. Moreover, GPs in urban areas appear to perform ultrasound exams more often compared to suburban and rural areas. Our finding contradicts other studies that illustrate geographical challenges as an incentive for using ultrasound in rural areas (e.g. Greenland, parts of Finland, Iceland, Norway, Scotland and Sweden) [12]. This may be less likely in Switzerland due to the ageing GPs and the shortfall of young successors. Indeed, with an average age of 53, GPs wishing for early retirement have trouble nowadays finding replacement to take over their individual practices in peripheral regions [22]. Under such circumstances, GPs are left overwhelmed and might have little to no time to perform ultrasound exams. Our study also shows that GPs in group practices perform ultrasound more often compared to those in individual practices. This finding is probably due to the efficiency of GP practices. To illustrate, group practices more often own an ultrasound machine compared to individual practices [11]. More so, our survey results show that most of the GPs working in group practices (92%) indicate the availability of the machine as a motivation to use ultrasound.

The overall mean of ultrasound productivity (129 exams per physician-year) in our study is less than indicated in a systematic review, which reported a range of between 131 and 601 exams per GP annually [23]. Many factors have possibly contributed to this difference. Indeed, the number of exams performed by our GPs varied widely, with 49% of the GPs using ultrasound less frequently than weekly. Nevertheless, we were still able to include light users in our data collection. The lower overall productivity compared to the individual years is probably caused by unproductive years with zero exams that are only taken into account in the overall value. In addition, the higher overall number of GPs compared to the individual years is probably caused by GPs that join during the 15-year period but are not visible in the yearly values because of retiring GPs.

Other reasons behind the variation in utilization can potentially be attributed to the allocation of tasks between GPs and specialists, the difference in ultrasound experience among GPs themselves, and the rather low proportion of GPs working full time in Switzerland [24]. These factors may result in the referral of patients to radiologists for ultrasound imaging rather than to other specialized GPs. Of course, the quantity and quality of an ultrasound exam also depend on the skills of the practitioner. As a rule, physicians in Switzerland have to provide an evidence of 500 fully performed and documented sonographies as pre-requisite for certification. Recertification is mandatory every 5 years; physicians are required to complete 50 credits of proven structured training and self-study, regardless of the level of their employment [25]. However, it remains unclear if these measures are enough for even a well-trained GP, to maintain competence and skills and ensure quality of scans amid low frequency of ultrasound use.

The current study provides an important view on the use of ultrasound in relation to patient’s morbidities. Our findings indicate that patients with multi-morbidities receive more ultrasound exams on average, but the study also shows that those with single morbidities have more exams per doctor visit. This finding from billing data is comparable to our survey results and previous studies, which suggest that ultrasound is mainly used for diagnostic purposes and less for therapeutic or treatment evaluation [2, 10]. On the other hand, our findings do not include all morbidities considering that morbidity could only be determined if medications were dispensed to patients during their GP encounters. Therefore, patients who were not dispensed medications, received it outside the GP’s practice, or were prescribed medications not conforming to the PCG model could not be accounted for in the analysis. In addition, our findings are based on the billing data of GPs in contract with the trust center, and do not account for medical encounters outside the billing group. Hence, these factors may have contributed to imprecise estimates in our findings, making it only possible to provide an overview of morbidities among patients receiving ultrasound, rather than establishing relationships between the two. Our study shows that female patients have more ultrasound exams and receive it mainly for the conditions of the abdomen and pelvis. This finding is consistent with previous studies in which females represented 58 to 60% of patients having ultrasound; chronic abdominal pain was reported as a primary indication for abdominal and pelvic exams [10, 26]. Compared to males, females had more exams for head and neck conditions and procedural guidance. These results are consistent to other studies; the reported indications for such exams included breast lump and pain, neck swelling and enlarged thyroid [26].

Comparable to other studies, our results show that abdominal ultrasound (68%) is the most frequent ultrasound exam performed by GPs in primary care [1, 7, 11, 12, 23, 26–28]. The study also demonstrates that male patients had more ultrasound exams for the abdomen (85.77%) compared to females (56.23%). However, this estimate must be weighed in light of the Tarmed classification system; ultrasound of abdomen includes exams that also investigate genito-urinary system in males, whereas an additional Tarmed category is allocated for genital investigation in females, therefore resulting in this discrepancy. The overall usage of ultrasound in the diagnosis of head and neck, abdominal, and pelvic conditions is equally reflected in billing data of utilization and self-reported indications. However, the use of ultrasound, for indications targeting vessels, musculoskeletal and soft tissues is lower among GPs in the billing data compared to those surveyed. This incomparability in usage between the two groups may suggest underreporting of the utilization of ultrasound by GPs, considering that the billing data only account for examinations that were eligible and submitted for remuneration.

The main clinical conditions indicated by GPs for the use of ultrasound in Swiss primary care correspond to the findings of previous studies [1, 7, 12, 23, 26–28]. Nevertheless, we found variations in the indication of ultrasound for some medical conditions, which may not be in line with recent guidelines and recommendations. For instance, the use of ultrasound for the diagnosis of acute appendicitis is not frequent among our surveyed GPs, although it is recommended in recent studies for suspected acute appendicitis in pediatric and adult patients [29, 30]. Similarly, a meta-analysis conducted in 2011 reported the superiority of ultrasound to radiography in the detection of pneumothorax [31]; however, our survey findings show that only 35% of GPs utilize it for this purpose. Further, the application of ultrasound in the diagnosis of fractures or for procedural guidance, particularly in musculoskeletal and soft tissue injections, is relatively low in spite of the diagnostic accuracy and the cost effectiveness of ultrasound imaging in primary care for such indications [32, 33]. It is possible that these conditions are not overly common across Swiss primary care and are more prevalent in emergency room settings, which may therefore explain this disparity.

Contrary to what was previously reported, the current study demonstrates that GPs indeed use ultrasound for gynecological conditions in Swiss primary care, yet at a lower frequency compared to other indications. This finding is equally reflected in the general reduction of the gynecological services provided by primary care physicians in Switzerland [34, 35].

The findings also suggest that some GPs are performing comprehensive ultrasound exams, targeting the diseases of the liver, spleen, and kidneys, and comprising the detection of rib fractures, evaluation of the prostate, palpable neck masses, abscesses and cysts. Others appear to be better focused on specific indications that provide targeted clinical answers, such as free fluid in the abdomen, kidney congestion, cholecystolithiasis and bladder-filling conditions (see Supplementary Table 4). These findings may be interpreted in light of the recently introduced changes in ultrasound education and training certification in Switzerland [36]. In the beginning of 2018, SGUM started awarding point of care ultrasound (POCUS) certification course to promote and improve the utilization of ultrasound among physicians, and reduce hazards associated with adoption of the technique by users with inadequate experience, training and skills [36, 37] . Prior to this, GPs and specialists alike, had to undergo extensive certification process in order to perform ultrasonographic examination that encompasses an entire anatomical region, a physiological unit or an organ, which meant longer examination time. Alternatively, the POCUS module offers fewer exams for certification and endorses clinical questions to allow physicians to answer these questions in a focused and efficient manner [36]. It is too early to associate the introduction of POCUS certification with our longitudinal results, but our findings may be used as benchmark for future research to evaluate the impact of POCUS certification on the variety of ultrasound examinations and the ultrasound productivity in general practice.

In line with previous studies, our survey findings emphasize the general view of users that faster diagnosis and earlier treatment are the main reasons for utilizing ultrasound in primary care [2, 7]. Conversely, GPs selected financial benefits among the least important reasons for the utilization. These findings may suggest that the use of ultrasound in primary care is not heavily dependent on the reimbursement, which contradicts other studies that illustrate the financial aspect as a barrier for the use of ultrasound in primary care [12, 20]. Yet, due to the descriptive nature of the survey, we can neither confirm these findings, nor determine to which extent the utilization of ultrasound in primary care is dependent on certification or reimbursement.

### Strengths and limitations

The strength of this study is underscored by the broad agreement of its findings across both billing and cross-sectional data. However, like most of the observational studies, the results we herein report should be considered in the light of some limitations. Due to the short time frame of the study, the survey group was limited to SGUM participants and restricted to courses within the data collection period. Similarly, the billing group included only GPs in Central Switzerland, hence precluding the generalizability of our results. In addition, the decentralization of SGUM courses made it difficult to estimate the response rate of the survey participants. Lastly, we were unable to acquire detailed data from patients without any ultrasound exam, limiting our capability to calculate predictors among all patients.

### Implications for future research

The current study provides an overview of the clinical uses of ultrasound and the general views of GPs about this use; this in turn stimulates series of questions for further investigation. Indeed, research is warranted to examine potential reasons underlying the variation in the utilization and the indication of ultrasound. It remains to be clarified if variation is associated with quality and effectiveness of ultrasound. In that respect, it will be important to investigate if POCUS certification with its restriction to focused indication will affect these indicators. Qualitative research may be needed to identify barriers and facilitators for ultrasound use, and to explore the perspectives of general practitioners on ultrasound training and their motivation for certification. Research in these areas is deemed essential to inform stakeholders about possible changes for the promotion and the integration of ultrasound in primary care, and for the benefit of patients and the health care system alike.

## Conclusion

The use of ultrasound is high among general practitioners, and it covers a wide range of clinical indications. Ultrasound is utilized primarily in the diagnosis of clinical indications of the abdomen, and more often for female than male patients.

## Supplementary information

**Additional file 1: Table S1.** Distribution of patients with single morbidity, by-morbidity type, mean of US exams and doctor visits over 15-year period. **Table S2.** Yearly distribution of US exams, frequency of patients and GPs and US productivity. **Table S3.** Views of GPs on different aspects of US use. **Table S4.** Frequency of GPs presently using or anticipating to use ultrasound for the targeted indications of POCUS.

## Data Availability

The dataset is not publicly available due to the sensitivity of the data, but it is available from the corresponding author on reasonable request.

## Abbreviations

POCUS: Point-of-Care ultrasound
GP: General Practitioner
SGUM: Swiss Society of Ultrasound in Medicine
PCG: Pharmacy-based Cost Group
ATC: Anatomical Therapeutic Chemical Classification System

## References

[1] Decrey H, Verdon F, Burnand B, Pécoud A, Burnier M (1998). Evaluation of the use of ultrasonography in primary care. Eur J Pub Health.

[2] Bhagra A, Tierney DM, Sekiguchi H, Soni NJ (2016). Point-of-care ultrasonography for primary care physicians and general internists. Mayo Clin Proc.

[3] Smallwood N, Dachsel M (2018). Point-of-care ultrasound (POCUS): unnecessary gadgetry or evidence-based medicine?. Clin Med.

[4] Ultrasound Guidelines: Emergency, Point-of-Care and Clinical Ultrasound Guidelines in Medicine. Ann Emerg Med. 2017;69(5):e27–54. 10.1016/j.annemergmed.2016.08.457. 10.1016/j.annemergmed.2016.08.457 28442101

[5] Bono F, Campanini A (2007). The METIS project for generalist ultrasonography. J Ultrasound.

[6] Robinson L, Potterton J, Owen P (1997). Diagnostic ultrasound: a primary care-led service?. Br J Gen Pract.

[7] Genc A, Ryk M, Suwała M, Żurakowska T, Kosiak W (2016). Ultrasound imaging in the general practitioner’s office – a literature review. J Ultrason.

[8] Hahn RG, Davies TC, Rodney WM (1988). Diagnostic ultrasound in general practice. Fam Pract.

[9] Wordsworth S, Scott A. Ultrasound scanning by general practitioners: is it worthwhile? J Public Health Med. 2002;24(2):88–94. 10.1093/pubmed/24.2.88 12141591

[10] Chan VSP, Piterman L, McCall L (1999). Use of clinical ultrasonography in an Australian suburban family practice: its indications and findings. Hong Kong Practitioner.

[11] Aakjær Andersen C, Jensen MBB, Toftegaard BS, Vedsted P, Harris M, Örenäs research group (2019). Primary care physicians’ access to in-house ultrasound examinations across Europe: a questionnaire study. BMJ Open.

[12] Mengel-Jørgensen T, Jensen MB (2016). Variation in the use of point-of-care ultrasound in general practice in various European countries. Results of a survey among experts. Eur J Gen Pract.

[13] Bundesamt für Statistik (2017). Europäischer Urbanisierungsgrad 2011 (DEGURBA - Eurostat).

[14] TARMED Online Browser [Internet]. https://www.tarmed-browser.ch/de. Accessed 12 Feb 2020.

[15] WHO Collaborating Centre for Drug Statistics Methodology (2018). Guidelines for ATC classification and DDD assignment 2019.

[16] Lamers LM, van Vliet RCJA (2004). The pharmacy-based cost group model: validating and adjusting the classification of medications for chronic conditions to the Dutch situation. Health Policy.

[17] Mercer S, Fischbacher-Smith D, Furler J, Sanci L, Moffat K, de Silva D (2016). Safer primary care: caring for people with multiple conditions. Technical series on safer primary care.

[18] Siu T, Chau H, Myhre D (2013). Bedside ultrasonography performed by family physicians in outpatient medical offices in Whitehorse, Yukon. Can J Rural Med.

[19] Ailon J, Mourad O, Nadjafi M, Cavalcanti R (2016). Point-of-care ultrasound as a competency for general internists: a survey of internal medicine training programs in Canada. Can Med Educ J.

[20] Iacob M (2016). Evidence at the point of care ultrasonography in family medicine. World Book of Family Medicine- Iberoamericana.

[21] Tandjung R, Hanhart A, Bartschi F, Keller R, Steinhauer A, Rosemann T (2015). Referral rates in Swiss primary care with a special emphasis on reasons for encounter. Swiss Med Wkly.

[22] Cribari M, Holzer BM, Battegay E, Minder CE, Zimmerli LU. What makes internal medicine attractive for the millennial generation? A survey of residents in internal medicine in Switzerland. Swiss Med Wkly. 2018;148:w14696. 10.4414/smw.2018.14696 30552857

[23] Andersen CA, Holden S, Vela J, Rathleff MS, Jensen MB (2019). Point-of-care ultrasound in general practice: a systematic review. Ann Fam Med.

[24] Jakob J, Cohidon C, Cornuz J, Selby K (2018). Participation in medical activities beyond standard consultations by Swiss general practitioners: a cross-sectional study. BMC Fam Pract.

[25] Schweizerische Gesellschaft für Ultraschall in der Medizin [Internet]. https://www.sgum.ch. Accessed 20 Apr 2020.

[26] Alamri AF, Khan I, Baig MIA, Iftikhar R (2014). Trends in ultrasound examination in family practice. J Fam Community Med.

[27] Birtwhistle RV, Sauerbrei EE (1983). Ultrasonography in the diagnosis of gallbladder disease. Can Fam Physician.

[28] Speets AM, Hoes AW, van der Graaf Y, Kalmijn S, de Wit NJ, van Swijndregt ADM (2006). Upper abdominal ultrasound in general practice: indications, diagnostic yield and consequences for patient management. Fam Pract.

[29] Mostbeck G, Adam EJ, Nielsen MB, Claudon M, Clevert D, Nicolau C (2016). How to diagnose acute appendicitis: ultrasound first. Insights Imaging.

[30] Debnath J, George RA, Ravikumar R (2017). Imaging in acute appendicitis: what, when, and why?. Med J Armed Forces India.

[31] Ding W, Shen Y, Yang J, He X, Zhang M (2011). Diagnosis of pneumothorax by radiography and ultrasonography: a meta-analysis. Chest..

[32] Frouzan A, Masoumi K, Delirroyfard A, Mazdaie B, Bagherzadegan E (2017). Diagnostic accuracy of ultrasound in upper and lower extremity long bone fractures of emergency department trauma patients. Electron Physician.

[33] Bee WW, Thing J (2017). Ultrasound-guided injections in primary care: evidence, costs, and suggestions for change. Br J Gen Pract.

[34] Cohidon C, Cornuz J, Senn N (2015). Primary care in Switzerland: evolution of physicians’ profile and activities in twenty years (1993-2012). BMC Fam Pract.

[35] Kringos DS, Boerma WGW, Hutchinson A (2015). Building primary care in a changing Europe. In: edited by series;40 OS.

[36] Knoblauch C, Canova C, Bauer W, Ganter C (2018). Der neue Fähigkeitsausweis POCUS. Schweizerische Ärztezeitung.

[37] Top 10 Health Technology Hazards for 2020 Executive Brief [Internet]. https://elautoclave.files.wordpress.com/2019/10/ecri-top-10-technology-hazards-2020.pdf.

