# Supplementary material for: The use of ultrasound in primary care: longitudinal billing and cross-sectional survey study in Switzerland
Source: BMC Fam Pract. 2020 Jul 1;21:127. doi: 10.1186/s12875-020-01209-7 (PMC7330951; doi:10.1186/s12875-020-01209-7)
Supplement: Supplementary file 1 — Additional file 1: Table S1. Distribution of patients with single morbidity, by-morbidity type, mean of US exams and doctor visits over 15-year period. Table S2. Yearly distribution of US exams, frequency of patients and GPs and US productivity. Table S3. Views of GPs on different aspects of US use. Table S4. Frequency of GPs presently using or anticipating to use ultrasound for the targeted indications of POCUS. [file 12875_2020_1209_MOESM1_ESM.docx]

# Supplementary Tables

| **Supplementary Table 1: Distribution of patients with single morbidity by morbidity type, mean of US exams and doctor visits over 15-year period** | | | |
| --- | --- | --- | --- |
| ***Type of Morbidity*** | **Patients**  n (%) | **US exams***  Mean (CI) | **Doctor visits**  Mean (CI) |
| Total | 11’399 | 1.70 (1.67 – 1.72) | 8.80 (8.64 – 8.96) |
| *Pain and Inflammation* | 6’099 (53.50) | 1.64 (1.61 – 1.67) | 9.08 (8.84 – 9.31) |
| *Peptic Ulcer* | 1'707 (14.97) | 1.83 (1.76 – 1.91) | 7.69 (7.37 – 8.02) |
| *Hypertension* | 550 (4.82) | 1.67 (1.57 – 1.78) | 9.78 (9.09 – 10.48) |
| *Vascular Diseases* | 426 (3.74) | 1.73 (1.58 – 1.87) | 9.86 (8.87 – 10.86) |
| *Rheumatoid Arthritis* | 400 (3.51) | 1.76 (1.62 – 1.89) | 8.85 (8.13- 9.58) |
| *Respiratory Illness/Asthma* | 378 (3.32) | 1.62 (1.48 – 1.75) | 8.29 (7.68 – 8.91) |
| *Depression* | 334 (2.93) | 1.69 (1.54 – 1.84) | 9.67 (8.85 – 10.48) |
| *Thyroid Disorders* | 270 (2.37) | 2.27 (2.04 – 2.49) | 8.16 (7.40 – 8.92) |
| *Pain* | 240 (2.11) | 1.59 (1.43 – 1.76) | 6.86 (6.03 – 7.68) |
| *Hyperlipidemia* | 231(2.03) | 1.84 (1.68 – 2.00) | 8.27 (7.44 – 9.09) |
| *Anxiety and Tension* | 197 (1.73) | 1.64 (1.45 – 1.83) | 6.81 (6.01 – 7.61) |
| *Cardiovascular* | 175 (1.54) | 1.69 (1.43 – 1.94) | 9.56 (7.82 – 11.30) |
| *Cystic Fibrosis* | 174 (1.53) | 1.58 (1.41 – 1.75) | 6.26 (5.56 – 6.96) |
| *Diabetes* | 72 (0.63) | 1.85 (1.42 – 2.27) | 11.35 (9.34 – 13.36) |
| *Psychotic illness* | 36 (0.32) | 1.64 (1.23 – 2.04) | 12.62 (7.45 – 17.78) |
| *Crohn's and Ulcerative Colitis* | 33 (0.29) | 1.60 (1.34 – 1.87) | 12.00 (6.46 – 17.54) |
| *Epilepsy* | 23 (0.20) | 1.91 (1.25 – 2.58) | 12.70 (6.72 – 18.67) |
| *Malignancies* | 23 (0.20) | 1.65 (1.23 – 2.08) | 10.48 (7.73 – 13.22) |
| *Parkinson's* | 12 (0.11) | 1.25 (0.96 – 1.54) | 9.42 (4.27 – 14.57) |
| *Gout* | 10 (0.09) | 1.00 (1) | 4.10 (2.40 – 5.80) |
| *Glaucoma* | 5 (0.04) | 1.40 (0.72 – 2.08) | 12.40 (1.14 – 23.66) |
| *Transplants* | 3 (0.03) | 1.34 (-0.10 – 2.77) | 25.67 (-13.64 – 64.97) |
| *HIV and AIDS* | 1 (0.01) | 2.00 (-) | 10.00 (-) |
| US: ultrasound; CI: Confidence Interval at 95% significance level  A dash (-) denotes that there were no observations under this group  *The total number of US exams for patients with single morbidity is 19’356 | | | |

| **Supplementary Table 2: Yearly distribution of US exams, frequency of patients and GPs and US productivity** | | | | |
| --- | --- | --- | --- | --- |
| **Year***  n=15 | **Patients †**  n= 67’180 | **GP †**  n= 104 | **US exams**  n=130’245 | **US productivity‡**  n= 96 |
|  | n (%) | n (%) | n (%) | n (mean; CI) |
| **2004** | 2’496 (3.7) | 39 (37.5) | 3’133 (2.4) | 39 (149.64; 82.67 - 216.62) |
| **2005** | 2’942 (4.4) | 41 (39.4) | 3’712 (2.9) | 41 (162.47; 95.09 - 229.85) |
| **2006** | 3’757 (5.6) | 48 (46.2) | 4’746 (3.6) | 48 (153.95; 95.05 - 212.85) |
| **2007** | 4’141 (6.2) | 52 (50.0) | 5’167(4.0) | 49 (152.05; 94.27 - 209.83) |
| **2008** | 6’242 (9.3) | 49 (47.1) | 8’199 (6.3) | 48 (161.75; 103.94 - 219.57) |
| **2009** | 6’004 (8.9) | 52 (50.0) | 7’980 (6.1) | 52 (152.07; 97.74 - 206.41) |
| **2010** | 6’693 (10.0) | 52 (50.0) | 9’011 (6.9) | 52 (161.09; 107.61 - 214.58) |
| **2011** | 7’367 (11.0) | 54 (51.9) | 9’909 (7.6) | 53 (159.33; 106.63 - 212.03) |
| **2012** | 6’878 (10.2) | 59 (56.7) | 9’156 (7.0) | 57 (152.04; 102.45 - 201.64) |
| **2013** | 6’771 (10.1) | 67 (64.4) | 8’839 (6.8) | 66 (152.49; 107.96 - 197.01) |
| **2014** | 8’092 (12.0) | 73 (70.2) | 10’827 (8.3) | 68 (159.16; 114.67 - 203.64) |
| **2015** | 9’409 (14.0) | 74 (71.2) | 13’093 (10.1) | 70 (166.15; 122.25 - 210.05) |
| **2016** | 10’030 (14.9) | 79 (76.0) | 14’042 (10.8) | 75 (159.79; 118.25 - 201.33) |
| **2017** | 9’916 (14.8) | 83 (79.8) | 13’645 (10.5) | 78 (155.42; 115.20 - 195.64) |
| **2018** | 6’622 (9.9) | 79 (76.0) | 8’786 (6.7) | 76 (148.45; 108.01 - 188.90) |
| US: ultrasound; CI: Confidence Interval at 95% significance level  *2018 data is up to 30 September 2018  † Total percentage is more than 100  ‡ The overall mean of US productivity is 128.72 for 96 GPs, the mean of US exams per year is 9937.24; n = number of GPs for that specific year | | | | |

| **Supplementary Table 3: Views of GPs on different aspects of US use** | | | |
| --- | --- | --- | --- |
|  | **Present use*** | **Anticipated use** | |
|  |  | Current users* | Non-users* |
|  |  |  |  |
| **General Reasons Total** | **53** | **5** | **6** |
| *Standard of practice*, n (%) | 41 (77) | 2 (40) | 2 (33) |
| *Availability of the machine at practice*, n (%) | 36 (68) |  | 3 (50) |
| *Reputation of the practice*, n (%) | 9 (17) | 2 (40) | 1 (17) |
| *Patient’s comfort and convenience*, n (%) | 30 (57) | - | 3 (50) |
| *Financial benefit*, n (%) | 9 (17) | 4 (80) | 3 (50) |
| *Faster diagnosis and earlier medical intervention*, n (%) | 48 (91) | - | 5 (83) |
| *Other* †, n (%) | 3 (6) | - | - |
| **Medical Purposes Total** | **54** | **3** | **7** |
| *Diagnostic*, n (%) | 54 (100) | - | 7 (100) |
| *Therapeutic*, n (%) | 22 (41) | 2 (67) | - |
| *Treatment evaluation*, n (%) | 23 (43) | 1 (33) | 1 (14) |
| **Relevance of US Skills to Practice Total** | **54** |  | **7** |
| *Very relevant*, n (%) | 27 (50) | - | 1 (14) |
| *Somewhat relevant*, n (%) | 24 (44) | - | 3 (43) |
| *Neither relevant nor irrelevant*, n (%) | 3 (6) | - | 2 (29) |
| *Somewhat irrelevant*, n (%) | - | - | 1 (14) |
| *Very irrelevant*, n (%) | - | - |  |
| **Comfort with US for Clinical assessment Total** | **54** |  | **7** |
| *Very comfortable*, n (%) | 20 (37) | - | - |
| *Somewhat comfortable*, n (%) | 24 (44) | - | 4 (57) |
| *Neither comfortable nor uncomfortable*, n (%) | 9 (17) | - | 2 (29) |
| *Somewhat uncomfortable*, n (%) | 1 (2) | - | 1 (14) |
| *Very uncomfortable*, n (%) | - | - | - |
| **Comfort with US for Guided procedures Total** | **16** |  | **1** |
| *Very comfortable*, n (%) | 4 (25) | - | - |
| *Somewhat comfortable*, n (%) | 9 (57) | - | - |
| *Neither comfortable nor uncomfortable*, n (%) | 2 (12) | - | 1 (100) |
| *Somewhat uncomfortable,* n (%) | 1 (6) | - | - |
| *Very uncomfortable,* n (%) | - | - | - |
| US: ultrasound; 54 GPs are presently using ultrasound, 7 GPs are not using ultrasound at their practices.  *Total percentage does not add to 100, GPs may have multiple selections.  † Other reasons included emergency (2), reduced cost for patients (1)  A dash (-) denotes that there were no observations under this group | | | |

**Supplementary Table 4: Frequency of GPs presently using or anticipating to use ultrasound for the targeted indications of POCUS**

| **POCUS targeted indications** | **Present use***  n=54 | **Anticipated use*** | | |
| --- | --- | --- | --- | --- |
|  |  | Current users  n=17 | Non-users  n=7 | |
|  | n (%) | n (%) | | n (%) |
| Pericardial effusion | 33 (61) | 4 (24) | | 6 (86) |
| Pneumothorax | 19 (35) | 10 (59) | | 3 (43) |
| Pleural effusion | 42 (78) | 5 (29) | | 4 (57) |
| Cholecystolithiasis | 49 (91) | 4 (24) | | 7 (100) |
| Cholecystitis | 46 (85) | 6 (35) | | 6 (86) |
| Free fluid in the abdomen | 50 (93) | 4 (24) | | 7 (100) |
| Kidney congestion | 50 (93) | 4 (24) | | 5 (71) |
| Abdominal aortic aneurysm | 42 (78) | 6 (35) | | 5 (71) |
| Bladder filling condition | 49 (91) | 2 (12) | | 7 (100) |
| Deep venous thrombosis | 28 (52) | 8 (47) | | 1 (14) |
| US for guided procedures | 14 (26) | - | | - |
| US for pain management | 4 (7) | - | | - |
| Targeted indications of POCUS according to SGUM POCUS education module  *Total percentage does not add to 100, many GPs perform more than one US type  A dash (-) denotes that there were no observations under this group | | | | |
